# Climate change impacts and the reshaping of Canadian viticulture

**DOI:** 10.1016/j.isci.2025.111941

**Published:** 2025-02-26

**Authors:** Massimiliano N. Lippa, Paolo Tarolli, Eugenio Straffelini

**Affiliations:** 1 Department of Land, Environment, Agriculture, and Forestry, University of Padova, 35020 Legnaro, Italy

**Keywords:** Climatology, Environmental science, Agricultural science, Land use

## Abstract

Shifting climate patterns across wine-growing areas of Ontario, British Columbia, Nova Scotia, and Quebec are driving the development of new viticultural potential within established Canadian wine regions. Changing trends of critical climatic variables and indices, such as near-surface temperature (NST) and growing degree days, indicate that growing conditions are changing. This research assesses NST and seasonal precipitation trends from 1994 to 2100 for Canadian viticulture, focusing on the primary established growing regions. Using multi-model CMIP6 spatial-temporal averages from the NEX-GDDP-CMIP6 dataset available on Google Earth Engine, this research aims to understand future NST and seasonal precipitation trends with climate scenarios SSP245 and SSP585 and discuss possible effects on viticulture on a near-term (2015–2050) and long-term (2050–2100) basis. Minimum, average, and maximum NST trends demonstrated statistically significant increases across all regions, with similar increasing precipitation trends across the growing season. Increasing trends, especially trends of extreme temperature, can all influence grape quality and, ultimately, wine quality. Outcomes suggest warmer growing climates, which may benefit wine producers, but the increasing frequency of extreme climate-change-related events such as drought, heatwaves, or extreme rainfall suggests potential future challenges that will require careful management.

## Highlights

• Growing conditions are shifting in Canadian viticultural areas
• Degree days trends are projected to increase across SSP245 and SSP585 pathways
• Growing seasons are becoming warmer and wetter, with some variability
• Shifting growing climates may allow for the use of new grape varieties

## Graphical abstract

**Figure:**
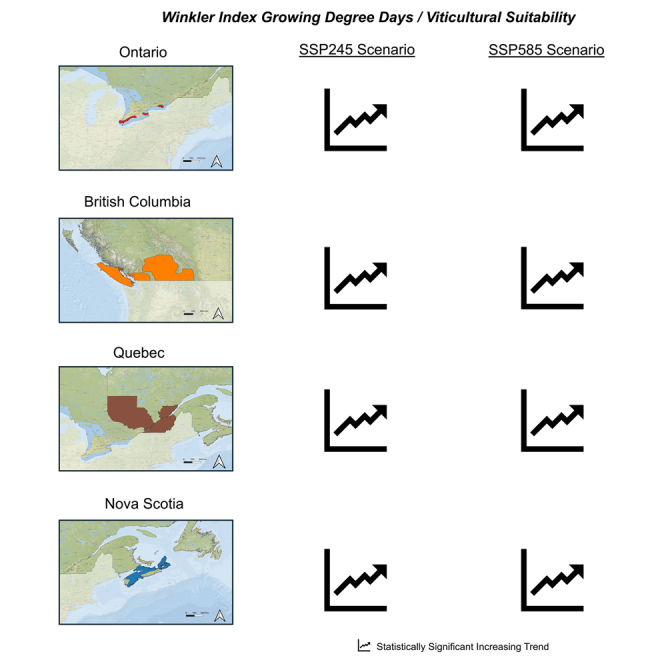

## Introduction

Favorable climates within some regions of Canada have facilitated the establishment of a thriving viticultural industry. Established wine-producing areas are primarily concentrated in the Canadian provinces of Ontario and British Columbia. Together, both provinces make up about 98% of the total wine production in Canada.^1^ However, other areas have been established in Quebec and Nova Scotia. Canadian wine-producing areas lie between the latitudes of 41 and 50°N, with wine production and its related industries contributing an estimated CAN$11 billion to the national economy.^2^^,^^3^

Canada is considered a cool-climate wine-producing nation. The growing season across the four provinces typically lasts from April to October.^4^^,^^5^^,^^6^ Vinifera that characterize Canadian wine production are typically of European descent (*Vitis vinifera*) or hybridized cultivars.^7^ Grape varieties such as Chardonnay, Riesling, and Pinot Noir are widely used by winemakers. According to the Köppen climate classification system, wine-growing areas within Ontario, Quebec, and Nova Scotia are classified as humid continental climates (Dfa and Dfb), whereas wine production areas in British Columbia stretch across both oceanic (Cfb) and humid continental (Dfa and Dfb) climate classifications.^8^

Wine and climate are inextricably tied to each other. A common phrase used often by those in the industry, “wine is grown, not made,” alludes to the importance of terroir and climate in determining the quality of the final bottle.^9^ Temperature and precipitation are among the most critical climate factors in creating a favorable terroir.^10^^,^^11^^,^^12^ Temperature is also important in grape phenology.^13^^,^^14^ Given the relatively narrow range of climate conditions that are needed to ensure viticultural success, alteration in critical growing climate components, such as temperature and precipitation, can have an influence on the long-term viability of commercial viticulture.^4^^,^^10^^,^^13^^,^^15^

According to *Canada’s Changing Climate Report* provided by Natural Resources Canada, Canadian temperatures are projected to increase.^16^ Concurrently, overall precipitation is also expected to increase, with decreases occurring in southern parts of the nation during the summer months.^16^ Benefits of such trends may include the cultivation of grape varieties once only found in warmer wine-producing nations, which may allow for commercial entry of Canada into new wine markets.^13^^,^^17^^,^^18^ However, changing climates, higher frequency of droughts, heatwaves, extreme storms, and increased pest pressure will provide significant barriers to viticultural success.^13^^,^^19^^,^^20^^,^^21^^,^^22^^,^^23^

Among the literature addressing Canadian viticulture, little addresses and compares the future climate impacts across all established growing areas within the four primary Canadian wine-producing provinces, tending to take a regional perspective instead. Existing literature primarily covers wine-producing areas in Ontario and British Columbia.^4^^,^^5^^,^^24^^,^^25^^,^^26^ Some literature of similar nature does exist for Quebec, as exemplified by Roy et al. (2017) and Jones (2018).^27^^,^^28^ Unlike the three other provinces, a focus on wine-producing regions in Nova Scotia appeared to be sparse. To the best of the authors’ knowledge, existing literature on Canadian viticulture rarely addresses and compares future climate impacts among established growing areas within Canada’s four major wine-producing provinces in unison, tending instead to adopt a regional perspective. Therefore, a general overview of the Canadian wine industry is still missing, which is important for planning future productions in a climate change context that present both opportunities and challenges. This research could contribute to fill this gap by analyzing future trends of seasonal precipitation and minimum, average, and maximum near-surface temperature (NST) considering all primary viticultural areas of the four most important wine-producing Canadian provinces. This research uses multi-model CMIP6 spatial-temporal averages to assess three research questions: (1) Does growing season minimum, average, and maximum NST and seasonal precipitation trends from 1994 to 2100 indicate any increasing, neutral, or decreasing trends? (2) Do these trends vary in the bud burst, flowering, and veraison/harvest stages? (3) What impacts may these trends have in the near and long-term future? Ultimately, this research can contribute to provide a future perspective on Canadian wine production, along with relevant information for decision-makers and other stakeholders.

### Study areas

**Figure 1:**
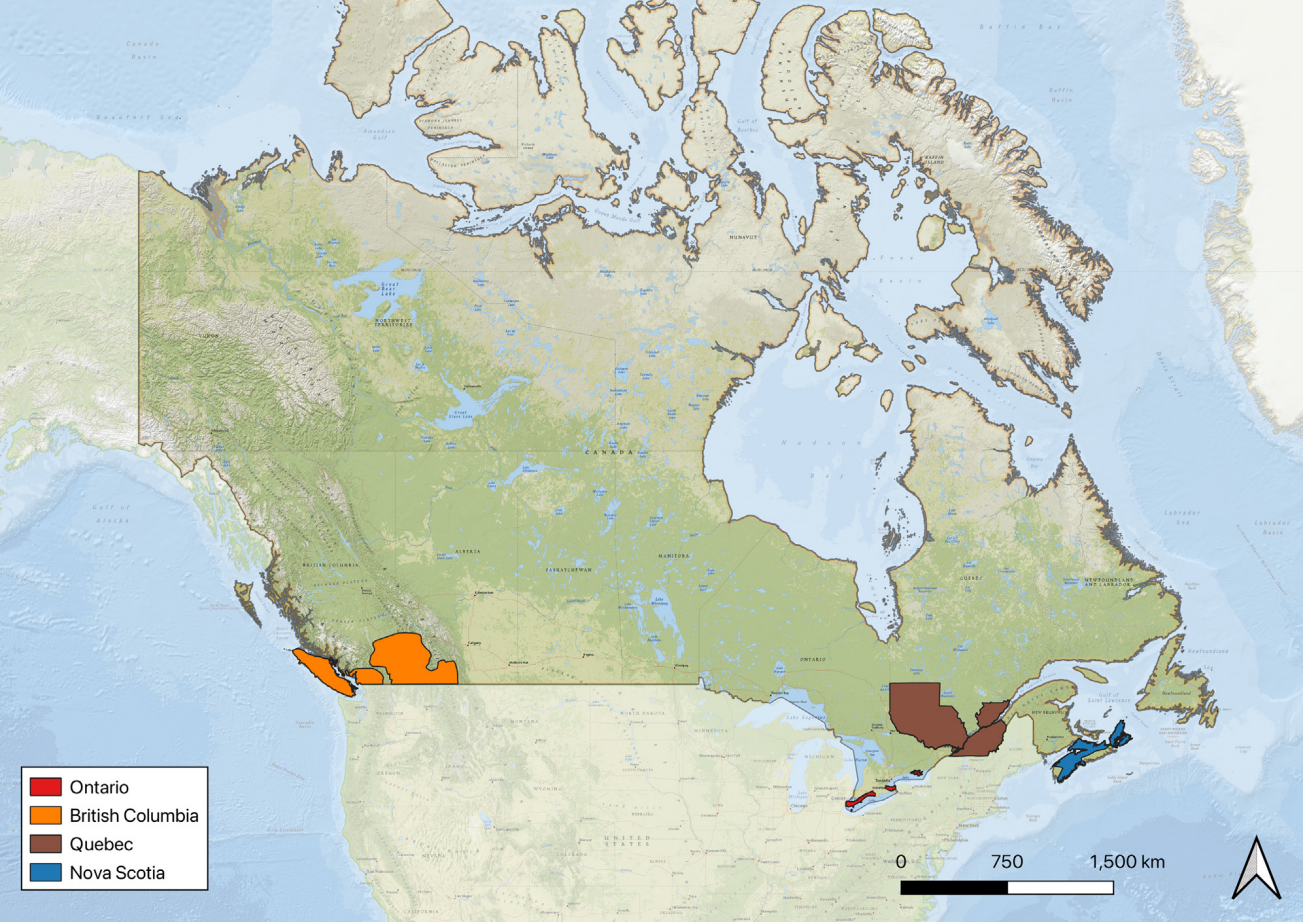
Study areas Major wine-grape-growing areas and their respective provinces of Canada adapted from maps and shapefiles provided by “*Wine map of Canada*” (2023), Jobin-Poirier et al. (2019), and Statistics Canada (2021).^19^^,^^29^^,^^30^

Wine-producing areas within four Canadian provinces, Ontario, British Columbia, Quebec, and Nova Scotia, were the focus of this research (Figure 1). Growing areas within these four provinces are Canada’s top wine producers in terms of production volume. Small wine-growing areas exist in other provinces; however, production quantity is limited, and their commercial value remains relatively local. This paper focuses only on established growing areas within Canada’s principal grape-wine-producing provinces.

## Results

### Near-surface temperature trends

**Figure 2:**
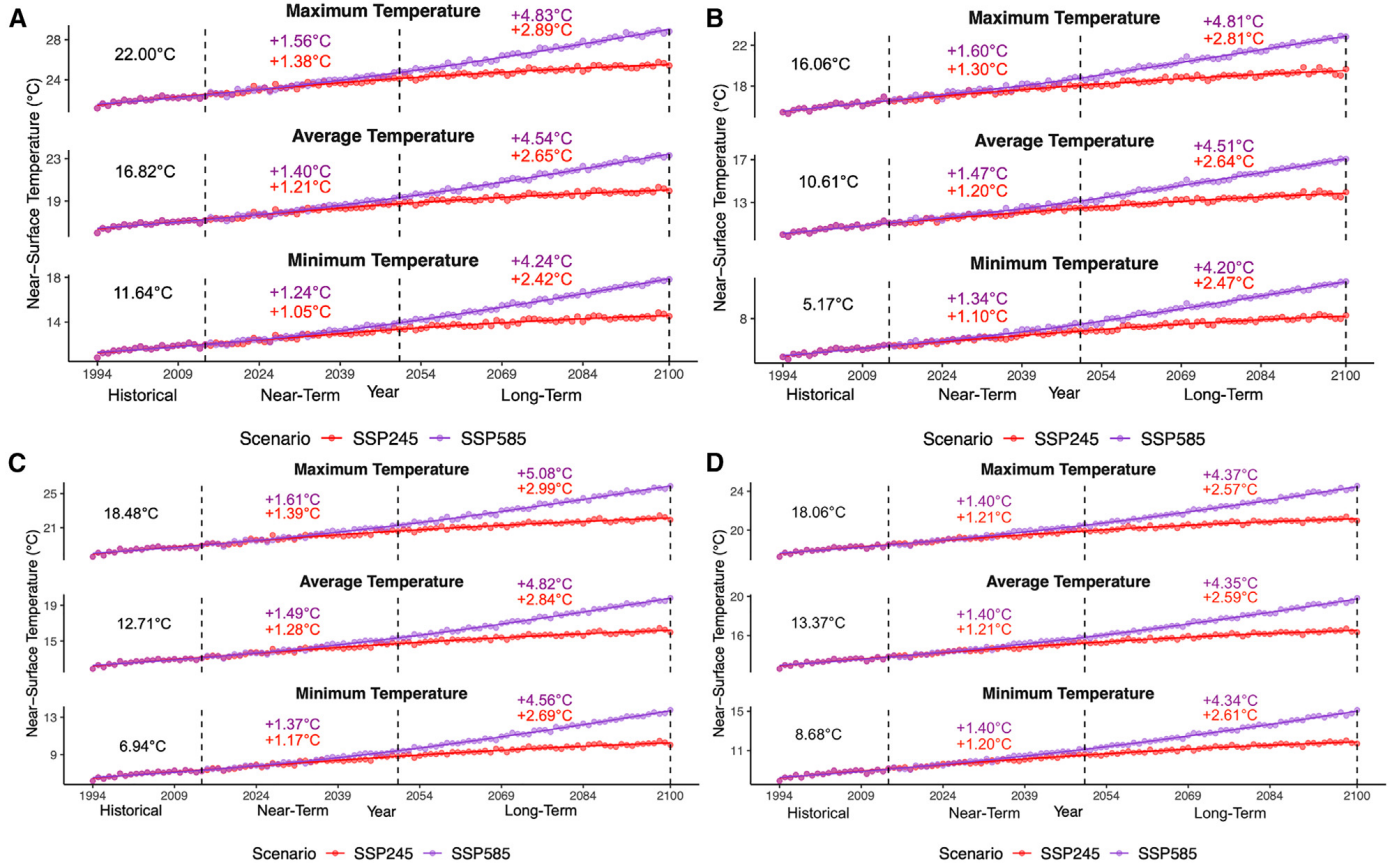
Growing season near-surface temperature (A–D) Historical, SSP245, and SSP585 multi-model CMIP6 yearly growing season near-surface temperature for (A) Ontario, (B) British Columbia, (C) Quebec, and (D) Nova Scotia. Each plot point represents a yearly spatial-temporal average across the region and growing season. In each plot, a locally estimated scatterplot smoothing (LOESS) curve is fit with a span of 0.75. The annotated values represent the change of near-surface temperature near-term (2015–2050) and long-term (2051–2100) in relation to the historical time period (1994–2014).

One aspect our research aims to understand is how minimum, average, and maximum NST change across the four temporal segments of interest. Across all regions of interest in each of the four provinces, positively sloped statistically significant average NST trends were observed in both scenarios (Figure 2; Table 1). In addition, average NSTs increased in the near-term and long-term in all growing regions in all provinces. Between 2015 and 2050 (near-term), average growing season NST in all areas never exceeded increases of 2°C from the historical baseline. Toward 2100, average growing season NST increased compared to the historical period ranging roughly above 2 and below 5°C.

**Table 1:** Near-surface temperature Mann-Kendall *p* values and Sens Slope

Scenario	Period	Number of years	Min temp Sens Slope	Min temp *p* value	Avg. temp Sens Slope	Avg. temp *p* value	Max temp Sens Slope	Max temp *p* value
**ON**								

SSP245	Growing season	107	0.03	≤0.001	0.04	≤0.001	0.04	≤0.001
SSP585	Growing season	107	0.06	≤0.001	0.07	≤0.001	0.07	≤0.001
SSP245	Bud burst	107	0.03	≤0.001	0.03	≤0.001	0.04	≤0.001
SSP585	Bud burst	107	0.06	≤0.001	0.06	≤0.001	0.06	≤0.001
SSP245	Flowering	107	0.03	≤0.001	0.03	≤0.001	0.04	≤0.001
SSP585	Flowering	107	0.06	≤0.001	0.07	≤0.001	0.07	≤0.001
SSP245	Veraison/harvest	107	0.04	≤0.001	0.04	≤0.001	0.04	≤0.001
SSP585	Veraison/harvest	107	0.07	≤0.001	0.07	≤0.001	0.08	≤0.001

**BC**								

SSP245	Growing season	107	0.03	≤0.001	0.04	≤0.001	0.04	≤0.001
SSP585	Growing season	107	0.06	≤0.001	0.07	≤0.001	0.07	≤0.001
SSP245	Bud burst	107	0.03	≤0.001	0.03	≤0.001	0.03	≤0.001
SSP585	Bud burst	107	0.05	≤0.001	0.06	≤0.001	0.06	≤0.001
SSP245	Flowering	107	0.03	≤0.001	0.04	≤0.001	0.04	≤0.001
SSP585	Flowering	107	0.07	≤0.001	0.07	≤0.001	0.08	≤0.001
SSP245	Veraison/harvest	107	0.04	≤0.001	0.04	≤0.001	0.04	≤0.001
SSP585	Veraison/harvest	107	0.07	≤0.001	0.07	≤0.001	0.07	≤0.001

**QC**								

SSP245	Growing season	107	0.04	≤0.001	0.04	≤0.001	0.04	≤0.001
SSP585	Growing season	107	0.07	≤0.001	0.07	≤0.001	0.08	≤0.001
SSP245	Bud burst	107	0.04	≤0.001	0.04	≤0.001	0.04	≤0.001
SSP585	Bud burst	107	0.07	≤0.001	0.07	≤0.001	0.07	≤0.001
SSP245	Flowering	107	0.04	≤0.001	0.04	≤0.001	0.04	≤0.001
SSP585	Flowering	107	0.06	≤0.001	0.07	≤0.001	0.07	≤0.001
SSP245	Veraison/harvest	107	0.04	≤0.001	0.04	≤0.001	0.04	≤0.001
SSP585	Veraison/harvest	107	0.07	≤0.001	0.08	≤0.001	0.08	≤0.001

**NS**								

SSP245	Growing season	107	0.03	≤0.001	0.03	≤0.001	0.03	≤0.001
SSP585	Growing season	107	0.06	≤0.001	0.06	≤0.001	0.06	≤0.001
SSP245	Bud burst	107	0.03	≤0.001	0.03	≤0.001	0.03	≤0.001
SSP585	Bud burst	107	0.06	≤0.001	0.06	≤0.001	0.06	≤0.001
SSP245	Flowering	107	0.04	≤0.001	0.04	≤0.001	0.04	≤0.001
SSP585	Flowering	107	0.06	≤0.001	0.07	≤0.001	0.07	≤0.001
SSP245	Veraison/harvest	107	0.04	≤0.001	0.04	≤0.001	0.04	≤0.001
SSP585	Veraison/harvest	107	0.07	≤0.001	0.07	≤0.001	0.07	≤0.001

Multi-model CMIP6 growing season, bud burst, flowering, and veraison/harvest near-surface temperature Mann-Kendall *p* values and Sens Slope for Ontario (ON), British Columbia (BC), Quebec (QC), and Nova Scotia (NS). (NS): Non-significant, Significant ≤0.05, Highly Significant ≤0.001

Considering the minimum and maximum temperatures over the growing season, our analysis shows statistically significant positive increases across the general growing season in the near and long-term. In all provinces during the growing season, near-term minimum NST increases exceeded 1°C, generally ranging above 1°C and below 2°C. In the long term, minimum temperature increases lay above 2°C and below 5°C. Maximum temperature changes followed a similar manner, with the long-term change of SSP585 in Quebec the only exception. Regardless of pathway, growing season maximum temperatures increased in all growing areas across all provinces and were generally consistent in the ranges of temperature increase both near- and long-term. Increasing maximum temperatures might prove problematic as extreme heat can cause desiccation or alter wine characteristics and profiles.

Temperature changes in generalized phenological (bud burst, flowering, veraison/harvest) temporal segments are also considered. Our research revealed statistically significant positive NST trends in the minimum, average, and maximums of the bud burst, flowering, and veraison/harvest phenological stages near- and long-term (see Figures S1–S3; Table 1). Near-term minimum, average, and maximum temperature changes from the historical baseline were relatively consistent in all phenological stages, with all increases relative to the historical baseline remaining below 2°C. Long-term NST (minimum, average, and maximum) increased above 2°C for both scenarios in all regions but never reached or surpassed 6°C. Overall, all growing regions in all provinces should expect to experience an increase in minimum, average, and maximum NSTs in the various phenological temporal segments set out in this research.

### Seasonal precipitation trends

**Figure 3:**
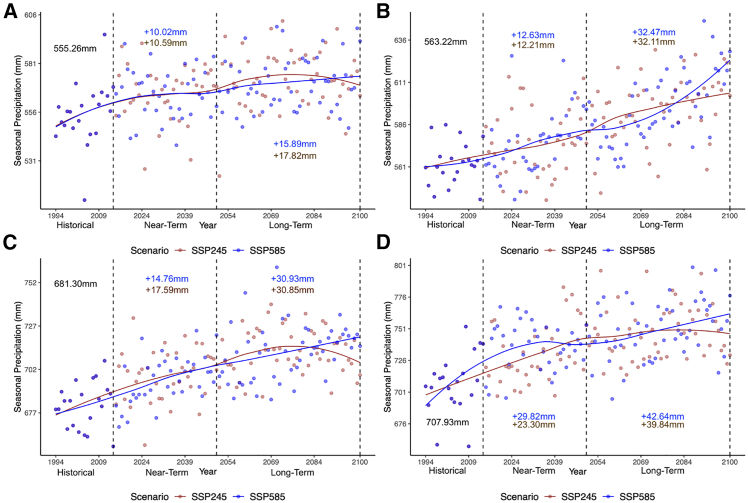
Growing season seasonal precipitation (A–D) Historical, SSP245, and SSP585 multi-model CMIP6 yearly growing season precipitation for (A) Ontario, (B) British Columbia, (C) Quebec, and (D) Nova Scotia. Each plot point represents a yearly spatial-temporal average across the region and growing season. In each plot, a locally estimated scatterplot smoothing (LOESS) curve is fit with a span of 0.75. The annotated values represent the change of near-surface temperature near-term (2015–2050) and long-term (2051–2100) in relation to the historical time period (1994–2014).

Seasonal precipitation trends were largely classified as statistically significant and increasing, with some regional and temporal variation (Figure 3; Table 2). All areas exhibited statistically significant increasing trends of precipitation during the growing season for both scenarios. In the near-term, growing season precipitation increases for both scenarios in all the wine-producing provinces ranged between 10 and 30 mm. Whereas, when assessing growing season precipitation changes closer to the end of the century, increases of precipitation were roughly between 15 and 43 mm depending on the scenario. Of the two more well-known wine-producing provinces in Canada, Ontario and British Columbia, British Columbia had larger increases of precipitation in both the near- and long-term. The remaining two provinces we evaluated, Quebec and Nova Scotia, also had notable increases of precipitation.

**Table 2:** Seasonal precipitation Mann-Kendall *p* values and Sens Slope

Scenario	Period	Number of years	Precipitation Sens Slope	Precipitation *p* value
**ON**				

SSP245	Growing season	107	0.21	≤0.001
SSP585	Growing season	107	0.18	≤0.001
SSP245	Bud burst	107	0.15	≤0.001
SSP585	Bud burst	107	0.24	≤0.001
SSP245	Flowering	107	−0.00	NS
SSP585	Flowering	107	−0.03	NS
SSP245	Veraison/harvest	107	0.06	NS
SSP585	Veraison/harvest	107	−0.00	NS

**BC**				

SSP245	Growing season	107	0.46	≤0.001
SSP585	Growing season	107	0.52	≤0.001
SSP245	Bud burst	107	0.28	≤0.001
SSP585	Bud burst	107	0.43	≤0.001
SSP245	Flowering	107	−0.07	≤0.001
SSP585	Flowering	107	−0.22	≤0.001
SSP245	Veraison/harvest	107	0.24	≤0.001
SSP585	Veraison/harvest	107	0.35	≤0.001

**QC**				

SSP245	Growing season	107	0.35	≤0.001
SSP585	Growing season	107	0.41	≤0.001
SSP245	Bud burst	107	0.22	≤0.001
SSP585	Bud burst	107	0.35	≤0.001
SSP245	Flowering	107	0.03	NS
SSP585	Flowering	107	−0.06	≤0.001
SSP245	Veraison/harvest	107	0.11	≤0.001
SSP585	Veraison/harvest	107	0.12	≤0.001

**NS**				

SSP245	Growing season	107	0.47	≤0.001
SSP585	Growing season	107	0.48	≤0.001
SSP245	Bud burst	107	0.26	≤0.001
SSP585	Bud burst	107	0.30	≤0.001
SSP245	Flowering	107	0.13	≤0.001
SSP585	Flowering	107	0.16	≤0.001
SSP245	Veraison/harvest	107	0.12	≤0.05
SSP585	Veraison/harvest	107	0.02	NS

Multi-model CMIP6 growing season, bud burst, flowering, and veraison/harvest seasonal precipitation Mann-Kendall *p* values and Sens Slope for Ontario (ON), British Columbia (BC), Quebec (QC), and Nova Scotia (NS).

(NS): Non-significant, Significant ≤0.05, Highly Significant ≤0.001.

Trends exhibited in the generalized phenological temporal segments were more variable than those found in the growing season (see Figures S4–S6; Table 2). All provinces had at least one statistically significant increasing trend for either bud burst, flowering, or the veraison/harvest temporal segments. Of the four provinces evaluated, Ontario had the lowest number of statistically significant trends identified, with all trends in the flowering and veraison/harvest statistically insignificant. During the bud burst in Ontario, increases in precipitation were projected to range from roughly 7 mm in the near-term and 12 to 16 mm in the long-term, depending on the scenario. Both the SSP245 and SSP585 trends of flowering and the SSP585 trend of veraison/harvest were slightly negative but statistically insignificant. In British Columbia, all precipitation trends were deemed statistically significant, with two significant decreasing trends identified in the flowering stage in the SSP245 and SSP585 scenarios. Rough declines between 4–6 mm in the near-term and 6 to 17 mm in the long-term were exhibited, again scenario dependent. Another statistically significant decreasing trend was observed during the flowering stage in Quebec under the SSP585 scenario, while the SSP245 scenario was insignificant. The remaining trends in Quebec’s bud burst and veraison/harvest temporal periods were statistically significant with positive slope values. All trends in Nova Scotia on the other hand, except for the veraison/harvest stage under the SSP585 scenario, were statistically significant with positive slope values, indicating increasing precipitation trends. Increases of precipitation were generally limited. However, precipitation increases can be problematic, especially in the end stages of the growing season, and can have several negative impacts on fruit quality.

### Winkler Index trends

**Figure 4:**
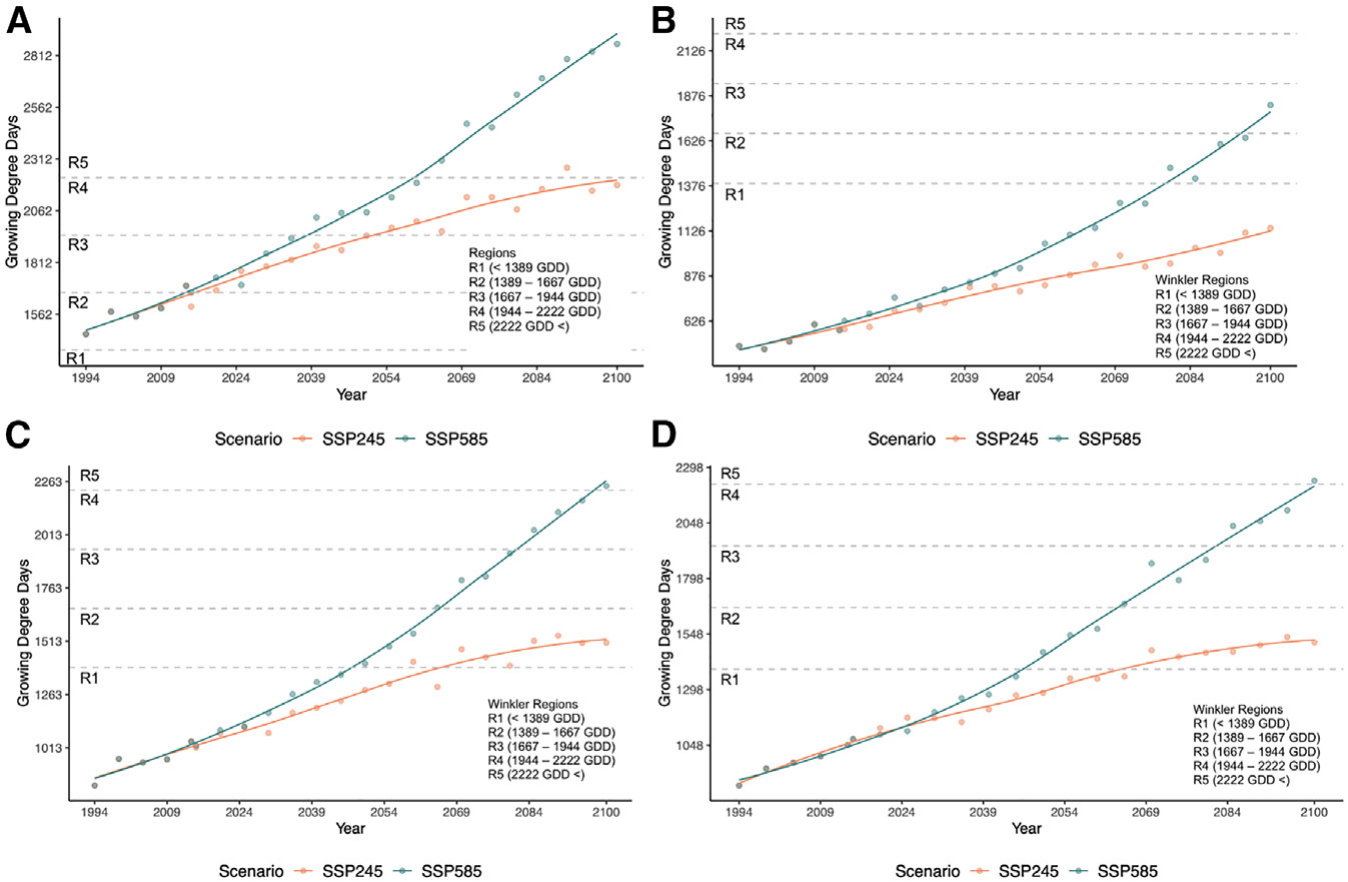
Winkler index (A–D) Winkler growing degree days for (A) Ontario, (B) British Columbia, (C) Quebec, and (D) Nova Scotia. Each plot point represents an average value from the seven subset models (Table 4) calculated regionally over the growing season. The data are shown at 5-year intervals and include the years 2014 and 2015 to account for the transition between historical and future scenarios. A locally estimated scatterplot smoothing (LOESS) curve with a span of 0.75 is fit to the data in each plot.

All trends of the Winkler Index, in both the SSP245 and SSP585 scenarios, indicated a positive and statistically significant trend for all provinces and their respective growing areas (Figure 4; Table 3). Therefore, all wine-producing areas in all provinces evaluated are expected to have an increasing number of growing degree days above the 10°C threshold moving toward the end of the century. All wine-producing provinces except for Ontario have initial Winkler values in the first Winkler region. The first Winkler values for Ontario begins in the second Winkler region. Moving into the middle century, British Columbia, Quebec, and Nova Scotia will have SSP245 Winkler values that remain in Winkler Region 1. The SSP245 scenario for Ontario will be on the edge of Region 3 moving into Region 4. For SSP585 trends in the middle of the century, British Columbia projects to be the only province of interest firmly remaining in Region 1, whereas Quebec and Nova Scotia will have entered Region 2. Ontario will be in Region 4. By the end of the century, the SSP245 scenario in British Columbia will remain in Region 1 while Ontario will occupy the degree day values around the threshold between Region 4 and Region 5. Quebec and Nova Scotia will both be in Region 2 under the SSP245 scenario by century’s end. Under the SSP585 scenario, growing degree days in Ontario will be classified as Region 5 while Quebec and Nova Scotia will begin to enter Region 5. By 2100, British Columbia is projected to have degree values that fall into the Region 3 categorization. The increasing Winkler trends observed indicate that across the study areas of interest, growing seasons are becoming more suitable for viticulture. Thus, wine-growing areas in the provinces of Ontario, British Columbia, Quebec, and Nova Scotia may be able to support the use of varieties typical of other global wine regions, like Nebbiolo, Grenache, or Grillo. However, there may also be a point where certain areas become too hot to support viticulture themselves, replicating the phenomena expected in current-day wine-growing areas of southern Europe.

**Table 3:** Winkler index Mann-Kendall *p* values and Sens Slope

Growing region	Scenario	Number of years	Sens Slope	*p* value
Ontario	SSP245 SSP585	23 23	34.53 66.07	≤0.001 ≤0.001
British Columbia	SSP245 SSP585	23 23	29.67 59.30	≤0.001 ≤0.001
Quebec	SSP245 SSP585	23 23	31.86 64.46	≤0.001 ≤0.001
Nova Scotia	SSP245 SSP585	23 23	29.63 62.32	≤0.001 ≤0.001

Winkler Mann-Kendall *p* values and Sens Slope for Ontario, British Columbia, Quebec, and Nova Scotia.

**Table 4:** NEX-GDDP-CMIP6 models and Winkler index subset

Model	Model provider	Country
*ACCESS-CM2*	Commonwealth Scientific and Industrial Research Organization	Australia
*ACCESS-ESM1-5*		
*BCC-CSM2-MR*	Beijing Climate Center	China
***CanESM5***	Canadian Center for Climate Modeling and Analysis	Canada
*CMCC-CM2-SR5*	Fondazione Centro Euro-Mediterraneo sui Cambiamenti Climatici	Italy
*CMCC-ESM2*		
***CNRM-CM6-1***	Center National de Recherches Météorologiques	France
*CNRM-ESM2-1*		
*EC-Earth3*	EC-Earth Consortium (Rossby Center, Swedish Meteorological and Hydrological Institute)	Sweden
*EC-Earth3-Veg-LR*		
*FGOALS-g3*	Chinese Academy of Sciences	China
*GFDL-CM4 (GR1)* *GFDL-CM4(GR2)* *GFDL-ESM4*	NOAA Geophysical Fluid Dynamics Laboratory	USA
*GISS-E2-1-G*	Goddard Institute for Space Studies, NASA	USA
*HadGEM3-GC31-LL*	Met Office, Hadley Center	UK
*HadGEM3-GC31-MM*		
*INM-CM4-8*	Institute for Numerical Mathematics	Russia
*INM-CM5-0*		
***IPSL-CM6A-LR***	Institut Pierre-Simon Laplace	France
*KACE-1-0-G*	National Institute of Meteorological Sciences (NIMS) and Korea Meteorological Administration (KMA)	South Korea
*KIOST-ESM*	Korea Institute of Ocean Science and Technology	South Korea
*MIROC-ES2L*	Japan Agency for Marine-Earth Science and Technology	Japan
***MIROC6***		
*MPI-ESM1-2-HR*	Max Planck Institute for Meteorology	Germany
***MPI-ESM1-2-LR***		
***MRI-ESM2-0***	Meteorological Research Institute	Japan
*NESM3*	Nanjing University of Information Science and Technology	China
*NorESM2-MM*	Norwegian Climate Center	Norway
***UKESM1-0-LL***	Met Office, Hadley Center	UK

The NEX-GDDP-CMIP6 models used in this research with Winkler index subset models bolded.^31^^,^^32^^,^^33^^,^^34^^,^^35^^,^^36^^,^^37^^,^^38^^,^^39^^,^^40^^,^^41^^,^^42^^,^^43^^,^^44^^,^^45^^,^^46^^,^^47^^,^^48^^,^^49^^,^^50^^,^^51^^,^^52^^,^^53^^,^^54^^,^^55^^,^^56^^,^^57^^,^^58^^,^^59^^,^^60^^,^^61^ Minimum (*tasmin*), average (*tas*), maximum temperature (*tasmax*), and precipitation (*pr*) outputs were used at a resolution of 27,830 m (0.25 × 0.25°) for historical, SSP245, and SSP585 scenarios.

## Discussion

### Alignment with existing studies

The increase of growing season temperatures observed in our research follows other papers that have made similar findings. Shifts toward warmer growing conditions have already been observed in several established global wine-producing regions, as mentioned in Jones et al. (2005), Cardell et al. (2019), Cabré & Nuñez (2020), Straffelini et al. (2023), and in Canada, as discussed by Holland & Smit (2014) and Hewer & Gough (2019).^15^^,^^25^^,^^62^^,^^63^^,^^64^^,^^65^ Further, projected future shifts toward warmer growing seasons, as our results suggest, fall in line with other works that project similar trends within Canada and abroad.^5^^,^^10^^,^^17^^,^^25^^,^^66^ A notable example is the United Kingdom as discussed by Nesbitt et al. (2022).^67^ Focusing on future suitability for Pinot Noir cultivation moving toward 2040, Nesbitt et al. (2022) also found an increase in growing season temperature.^67^ United Kingdom growing season temperatures were expected to shift from the average range of 13°C–15.7°C to a range of 13°C–17°C.^67^ Moreover, an increase in growing degree days was highlighted, which was very similar to the findings in this research.^67^ Similar outcomes to ours and Nesbitt et al. (2022) were also exhibited in Schultze et al. (2016) who studied the effects of warming on Michigan wine production.^67^^,^^68^ Michigan sits on the other side of the Detroit River and Lake Huron and is geographically very close to the wine-growing areas of southwestern Ontario. Schultze et al. (2016) concluded, similarly to the conclusions of this research, that increasing temperatures in Michigan could open future viticultural opportunities using new grape varieties.^68^ Trends identified in other emerging and cool-climate growing areas, such as the United Kingdom and Michigan, reinforce the findings of this research.

### Future trends and impacts on primary Canadian viticultural provinces

Increasing NSTs suggest a potential for growth of the Canadian wine industry. Across the growing areas in all regions, observed is increasing suitability according to Winkler both near- and long-term, indicating that even within the next quarter century, varieties unused in Canadian wine-growing areas may have some potential for utilization. With new varieties comes new wine production styles novel to the Canadian wine industry. Further, declining suitability in other regions globally may create gaps in the international wine market that Canadian wineries could fill because of improved growing season conditions. All regions, scenario dependent, by both 2050 and 2100 will likely have growing conditions reflective of warmer growing areas of Europe and the United States. Thus, the ability to grow varieties typical of warmer European or American wine regions may be possible in Canada.

To quell expectation, potential climate impacts will also provide many barriers. According to our results, producers should expect growing climates that are warmer and wetter with some regional and temporal variation. However, undesirable climate phenomena such as increasing frequency of extreme storm events, drought, pest pressures, and heatwaves all have the potential to affect overall wine quality and quantity.^13^^,^^19^^,^^20^^,^^21^^,^^23^ Warmer climates may cause grapes to ripen faster, possibly altering alcohol content and flavor profiles, which is problematic for wine-growing regions that produce wines with specific stylistic profiles.^69^ Warmer temperatures may also alter the color of red wines by decreasing pigment production.^70^^,^^71^^,^^72^ High rainfall amounts, especially late in the growing season, can negatively impact overall fruit quality by diluting sugar levels and increasing the risk of disease introduction.^19^ Inversely, lack of precipitation, especially under heat stress, could result in desiccation of fruit and inhibition of photosynthesis and development and increase the concentration of unfavorable compounds.^11^^,^^62^^,^^73^^,^^74^^,^^75^^,^^76^^,^^77^

### Vineyard management in response to extreme minimum and maximum growing season temperatures

Increasing minimum and maximum growing season temperatures may require the implementation or development of alternative vineyard management techniques. First, vineyard management must consider local climate conditions and needs to help offset climate impacts. Wineries need to turn to adaptive and regenerative management practices to help offset climate impacts, including heatwaves, extreme storms, and harvest loss from extreme cold or frost, with short-term and longer-term solutions.^15^^,^^25^^,^^78^ Shorter-term practices used in Canada to minimize damage overwintering include burying vines under the soil or using intra-row fires to help increase vineyard temperature and prevent damage caused by extreme cold and frost.^15^ Considering the need for long-term solutions to minimize negative impacts from warming temperatures, the use of new grape varieties adapted to warmer growing temperatures may be one strategy.^10^^,^^15^^,^^74^ However, this is a costly burden, both monetarily and in time. Increasing temperatures, as our results show, may increase water stress and evapotranspiration demand in growing areas. To meet water needs, practices like irrigation, having been embraced in other wine regions with high water stress, may be required to help offset declines in precipitation during dry periods.^79^ In older wine-growing regions in the world, such as Spain, longer-term solutions to combat the impacts of drought and heatwaves involve the pruning and training of vines to reduce vine size and water need, with one example being gobelet systems.^80^ Gobelet systems have existed within viticulture since Roman times, but they reduce overall vine size, thus lowering yield and water demand.^80^ One challenge of this system is the extreme difficulty of harvesting mechanically, with the upside of increased drought resistance.^74^ Given the possibility of increased frequency of extreme climate events, like drought and heatwaves within Canadian viticultural provinces, there may be some potential to use similar systems.

### Possible future impacts of wildfire on Canadian viticulture

There may be a risk of more significant interaction between viticultural areas and wildfire in the future, with increased water stress and aridity resulting from increasing growing season maximum NST.^81^^,^^82^^,^^83^ However, this risk needs to be geospatially quantified further as there is a lack of literature covering wildfires and their interactions with viticulture in the Canadian context. Given past wildfire seasons, especially in 2023, viticultural areas in Nova Scotia, British Columbia, and Quebec are at elevated risk levels compared to wine-producing areas in Ontario. The worst-case scenario for many winemakers is the destruction of vineyards. Furthermore, wildfire smoke in one area can also have far-reaching impacts on wine production in other areas. Smoke plumes from wildfires can spread great distances.^84^^,^^85^ The exposure of wine grapes to smoke produced by wildfires can result in wine with undesirable smoky flavor characteristics, known as smoke taint.^86^^,^^87^ Given the distances smoke can spread, this may pose a risk for viticultural areas in Ontario located outside of significant wildfire zones. This paper does not explicitly cover the viticultural-wildfire interface in Canada. However, given the historical frequency and severity of past wildfire seasons nearby and within viticultural regions of interest, it certainly must be considered.

### Conclusion

This paper explores the future of Canadian viticulture regarding two critical climatic variables: NST and seasonal precipitation. NST and seasonal precipitation trends were evaluated for 1994–2100 within Canada’s four primary wine-producing provinces: Ontario, British Columbia, Quebec, and Nova Scotia. From the results obtained, a consensus of NST displayed that statistically significant trends are present across both SSP pathways during the growing season, bud burst, flowering, and veraison/harvest periods. Long-term precipitation trends indicated general increases in the growing season but were more variable in their results in phenological stages than NST. Thus, more research must be conducted to further clarify precipitation trends.

Statistically significant increasing growing degree day trends suggest warmer future growing conditions for Canadian wine production. This should be contextualized and supplemented with further research of known phenomena of changing climates, such as extreme storms, drought, and heatwaves and their interactions with viticulture. However, a generally warmer climate may promote the shift of current viticultural traditions toward cool-climate nations like Canada. Further regional studies at higher resolutions are needed to compare model outputs and trends. However, our results should be used to inform wine producers, quality alliance labels, and local administrations on what future conditions may be evolving toward to help create flexible, resilient, and adaptable management strategies.

### Limitations of the study

Although this work has attempted to clarify the potential future of Canadian viticulture under climate change scenarios, it is not exempt from limitations. A significant component not considered in this research is soil, such as type, characteristics, and variation across wine-growing areas. Climate, including NST and precipitation, are just two aspects of the encompassing "terroir" concept. Soil type influences phenology, water availability, and nutrient provisioning.^88^^,^^89^ Although the influence of soil on wine production is not entirely understood in all aspects, soil is undoubtedly an essential aspect of viticultural viability.^21^^,^^63^^,^^74^^,^^88^^,^^89^^,^^90^^,^^91^^,^^92^ Other important parameters in addition to soil, such as sunlight exposure, humidity, evapotranspiration, change in water balance, and extreme hot or cold days, have not been considered and are limitations of this research.

Short-term and current impacts are not considered as thoroughly in this paper, which primarily addresses the broader long-term impacts that climate change is likely to have on the Canadian wine industry. Significant present-day and short-term impacts currently include, but are not limited to, potential future pests like *Lycorma delicatula* (Spotted Lanternfly), frost damage in both fall and spring seasons, and vine damage caused by extreme cold.^4^^,^^6^ Short-term impacts are expected to present ongoing constraints to Canadian wine producers and must be the focus of ongoing research.

Climate models also provide limitations, especially when at the coarse resolutions used in this research. Climate models are imperfect, and one must heed caution. Complex processes dictate climate patterns and models may be unable to adequately account for these processes, which may limit the utility of our research.^93^^,^^94^ The resolution of GCM’s in the NEX-GDDP-CMIP6 dataset may not adequately capture nuance and variation of temperature and precipitation trends within smaller spatial scales (Table 4). Further, the average of multi-model values across space and time can reduce the variability provided by individual models. Comparison to other models, possibly regional models, may be needed to understand if the complexities of climate processes are better reflected in other models. More region-specific research will be needed to understand climate dynamics at localized scales.

Given the sheer size of Canada and each wine-growing area, it is challenging to simultaneously complete a high-quality nationwide assessment of future NST and precipitation trends, given the different resolutions and coverage areas of climate models. Comparing the outputs of GCMs with regional climate models may be needed to understand further the complexities of climate processes. Furthermore, research should focus on adaptive mitigation techniques, improving water usage, and reducing the impacts of extreme events on vineyards.

## Resource availability

### Lead contact

Further information and requests for resources should be directed to and will be fulfilled by the lead contact, Paolo Tarolli (paolo.tarolli@unipd.it).

### Materials availability

This study did not generate new materials.

### Data and code availability

• Data: data reported in this paper will be shared by the lead contact upon request.
• Code: this paper does not report original code.
• Additional information: any additional information required to reanalyze the data reported in this paper is available from the lead contact upon request.

## Acknowledgments

The work is supported by the Italian PNRR programme for PhD and by the Agritech. National Research Center and received funding from the European Union Next- GenerationEU (PIANO NAZIONALE DI RIPRESA E RESILIENZA (PNRR) – MISSIONE. 4 COMPONENTE 2, INVESTIMENTO 1.4 – D.D. 1032 17/06/2022, CN00000022). This manuscript reflects only the authors’ views and opinions. Climate scenarios used were from the NEX-GDDP-CMIP6 dataset, prepared by the Climate Analytics Group and NASA Ames Research Center using the NASA Earth Exchange and distributed by the NASA Center for Climate Simulation (NCCS). We acknowledge the World Climate Research Program, which, through its Working Group on Coupled Modeling, coordinated and promoted CMIP6. We thank the climate modeling groups for producing and making available their model output, the Earth System Grid Federation (ESGF), for archiving the data and providing access, and the multiple funding agencies that support CMIP6 and ESGF.

## Author contributions

Conceptualization, M.N.L., P.T., and E.S.; data curation, M.N.L; methodology, M.N.L and E.S.; formal analysis, M.N.L.; visualization, M.N.L.; writing—original draft, M.N.L.; writing—review & editing, M.N.L., P.T., and E.S.; project administration, P.T.; supervision, P.T.; resources, P.T.

## Declaration of interests

Paolo Tarolli is a member of the iScience Advisory Board.

## STAR★Methods

### Key resources table

**Table:** 

REAGENT or RESOURCE	SOURCE	IDENTIFIER
**Deposited data**		

NEX-GDDP-CMIP6: NASA Earth Exchange Global Daily Downscaled Climate Projections	Google Earth Engine	https://developers.google.com/earth-engine/datasets/catalog/NASA_GDDP-CMIP6 https://www.nccs.nasa.gov/services/data-collections/land-based-products/nex-gddp-cmip6

**Software and algorithms**		

R Studio (Version 2023.09.0)	The R Project for Statistical Computing	https://cran.r-project.org/
QGIS (Version 3.32.2-Lima)	QGIS Project (OSGeo)	https://www.qgis.org
Google Earth Engine	Google	https://earthengine.google.com
Google Sheets	Google	https://workspace.google.com/products/sheets/

### Method details

#### Near-surface temperature & seasonal precipitation data collection

This research evaluated minimum, average, and maximum near-surface temperature (NST) at 2m height and seasonal precipitation trends from 1994 to 2100 across four generalized temporal segments: the growing season (April 1st – October 31st), bud burst phase (April 1st – June 15th), flowering phase (June 16th – August 1st), and veraison/harvest phase (August 2nd – October 31st). Temporal ranges for these segments selected, were informed by several papers, particularly Shaw (2016), Shaw (2012), Hewer & Gough (2021), and Rayne & Forest (2016).^4^^,^^6^^,^^95^^,^^96^ Temperature was calculated by finding a multi-model CMIP6 spatial-temporal average across all areas of interest, whereas seasonal precipitation was determined by finding a regional daily average precipitation rate and multiplying by the number of days in our period of interest to acquire seasonal precipitation. While growing season and phenology undoubtedly vary by grape variety and climate, this research focuses on general trends to provide a comprehensive overview of Canadian viticulture. Climate data for wine-growing regions in Ontario, British Columbia, Quebec and Nova Scotia were averaged over a singular region of combined growing areas for each province and were not evaluated appellation by appellation.

Climate data was extracted as a multi-model value from 29 General Circulation Models (GCM’s) provided by the NEX-GDDP-CMIP6 datasets at a resolution of 27,830m (roughly 0.25 × 0.25°) and were accessed through the Google Earth Engine platform.^97^^,^^98^^,^^99^ HadGEM3-GC31-MM, included in the 29 models selected, lacks data for SSP245 but does contain historical and SSP585 data. Therefore, our SSP245 scenarios are run with 28 models, while the SSP585 are run with 29 models. The NEX-GDDP-CMIP6 bands utilized for this research were *tasmin* (daily minimum NST), *tas* (daily NST), *tasmax* (daily maximum NST) and *pr* (mean of daily precipitation rate).^97^^,^^98^^,^^99^ The NEX-GDDP-CMIP6 dataset comprises of 35 GCM’s that were run as part of the Coupled Model Intercomparison Project Phase 6 and are derived from the ScenarioMIP’s.^100^^,^^101^^,^^102^ For our research, we selected 29 models based on data availability in the dataset (Table 4). The NEX-GDDP-CMIP6 dataset has been both bias corrected and downscaled using the Bias-Correction Spatial Disaggregation (BCSD) method, respectively.^103^ Historical model simulations were corrected using cumulative distribution functions in relation to corresponding outputs of the Global Meteorological Forcing Dataset (GMFD) and spatially disaggregated to a 0.25° grid.^103^

Two Shared Socioeconomic Pathways (SSP) cataloged in the NEX-GDDP-CMIP6 datasets, SSP245 (1994–2100) and SSP585 (1994–2100), were used to model future near-surface temperature and seasonal precipitation trends across our regions of interest. The pathways link willingness to implement climate change mitigation measures to assumptions about global society, resource usage, and technological development.^102^ Data from 1994 to 2014 lies within CMIP6’s historical scenario. The historical designation pertains to CMIP6 and is not a designation fabricated for this research. In addition, the historical period was utilized to calculate differences for near-term (2015–2050) and long-term (2051–2100) time frames to better inform decision-makers on near-term and long-term trends. From 2015 onwards, data falls under SSP245 or SSP585 scenarios, according to CMIP6. SSP245 provides an intermediate assumption of emission levels by the end of 2100, whereas SSP585 is the highest emission level amongst the SSP pathways by the end of the century.^102^ Using two socioeconomic pathways differentiates future scenarios of the Canadian wine industry and helps to provide a more comprehensive analysis.

All near-surface temperature and seasonal precipitation data were collected using Google Earth Engine. It is a cloud-based multi-petabyte data provider that provides open access to geospatial and satellite datasets.^104^^,^^105^ Users can access satellite and satellite-based products through Python or JavaScript interfaces. Data can be acquired in various formats, including temporal and spatial data formats, and used with other products like QGIS.^104^^,^^105^

#### Winkler index

This research selected a subset of the seven most representative models (Table 4) from the NEX-GDDP-CMIP6 dataset for the calculation of the Winkler Index, previously tested and evaluated for Canada by Bourdeau-Goulet and Hassanzadeh (2021).^106^ Winkler Index values were calculated every 5 years, including 2014 and 2015, to account for the transition between the historical and future scenarios. First developed by Amerine and Winkler (1944), the Winkler Index indicates heat summation over the growing season.^96^^,^^107^^,^^108^^,^^109^ The index is commonly used across viticultural literature.^6^^,^^15^^,^^24^^,^^26^^,^^66^^,^^110^ Using daily average temperature from the growing season, the Winkler Index provides an indication of suitability for grape cultivation through the summation of growing degree days (GDD) above a base temperature of 10°C.^6^^,^^15^^,^^96^^,^^109^^,^^110^ Sustained average growing season temperatures above 10°C is typically the accepted temperature threshold for vines to begin phenology.^20^^,^^107^^,^^108^ The Winkler Index gives five categorizations of suitability moving from coolest to warmest temperature (°C), 1) Region I (<1389 GDD), 2) Region II (1389–1667 GDD), 3) Region III (1667–1944 GDD), 4) Region IV (1944–2222 GDD), 5) Region V (2222< GDD).^6^^,^^11^^,^^15^^,^^26^^,^^96^^,^^107^^,^^108^^,^^109^

(Equation 1) $$GDD=\sum_{April\;1st}^{October\;31st}\max\;\left({0,T}_{avg}-10\right)$$

### Quantification and statistical analysis

The Mann-Kendall trend test, a non-parametric test, was used to determine if the observed trends were statistically significant. Formulated by Mann (1945) and Kendall (1938), and later adapted by others, this test is a non-parametric monotonic trend test over a time interval of interest comparing later values with earlier ones.^111^^,^^112^^,^^113^ Well cited and used in wine-related climate research, as found in Jones (2018), Fraga et al. (2015) and Teslić et al. (2016), the Mann-Kendall test determines whether y-values increase or decrease over time.^28^^,^^114^^,^^115^ In addition, a slope analysis using the non-parametric Sen’s slope provides an understanding of the magnitude of near-surface temperature and seasonal precipitation trends detected by Mann-Kendall.^116^

A locally estimated scatterplot smoothing (LOESS) line with a default span of 0.75 was used to visualize near-surface temperature and precipitation trends.^117^ Line placement is done in a non-parametric weighted fashion with no *apriori* assumptions, resulting in a line moving through the central tendency of the data spread.^117^ At each point in the dataset of interest, neighboring “local” points are more meaningful than points further away and thus have more influence on the resulting line.^117^
