# Supplementary material for: Climate change impacts and the reshaping of Canadian viticulture
Source: iScience. 2025 Feb 26;28(3):111941. doi: 10.1016/j.isci.2025.111941 (PMC11976484; doi:10.1016/j.isci.2025.111941)
Supplement: Document S1. Figures S1–S6 [file mmc1.pdf]

iScience, Volume *28*

Supplemental information

Climate change impacts and the reshaping of Canadian viticulture

Massimiliano N. Lippa, Paolo Tarolli, and Eugenio Straffelini

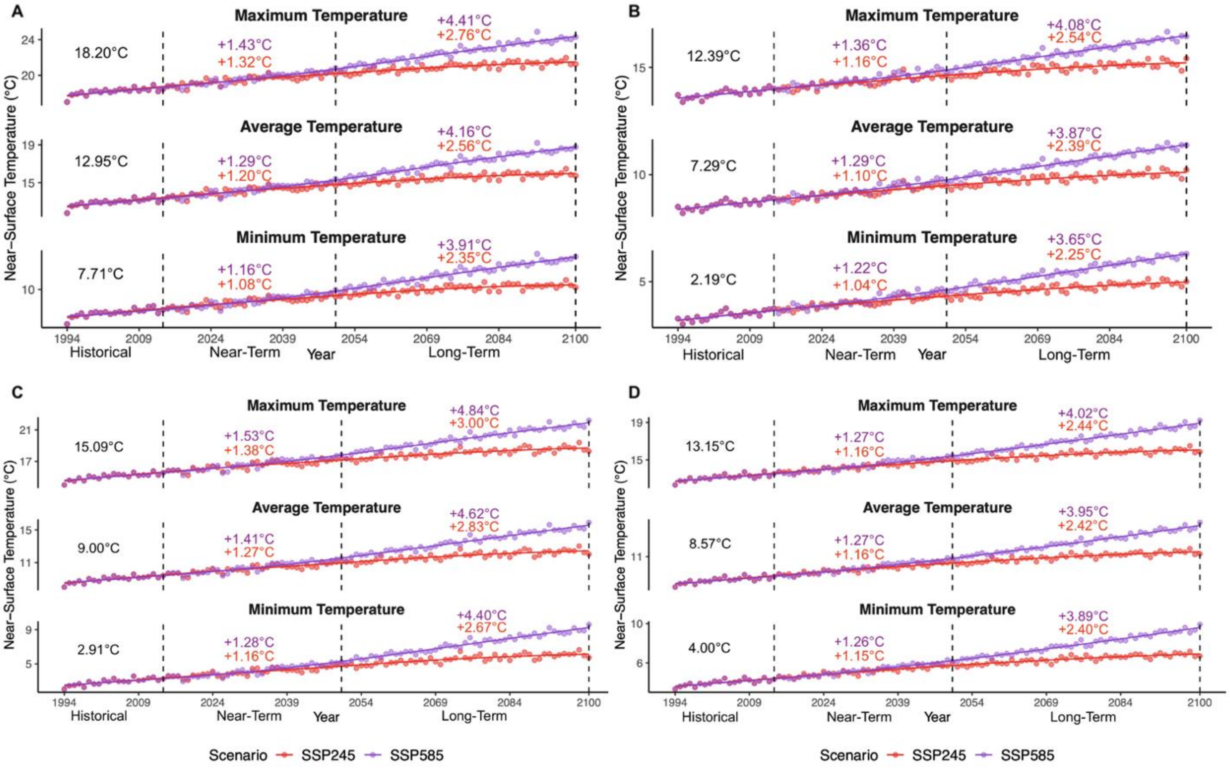

Figure S1: Bud Burst Near-Surface Temperature

Historical, SSP245, and SSP585 multi-model CMIP6 yearly bud burst near-surface temperature for A) Ontario, B) British Columbia, C) Quebec, and D) Nova Scotia. Each plot point represents a yearly spatial-temporal average across the region and bud burst period. In each plot, a locally estimated scatterplot smoothing (LOESS) curve is fit with a span of 0.75. The annotated values represent the change of near-surface temperature near-term (2015-2050) and long-term (2051-2100) in relation to the historical time period (1994-2014).

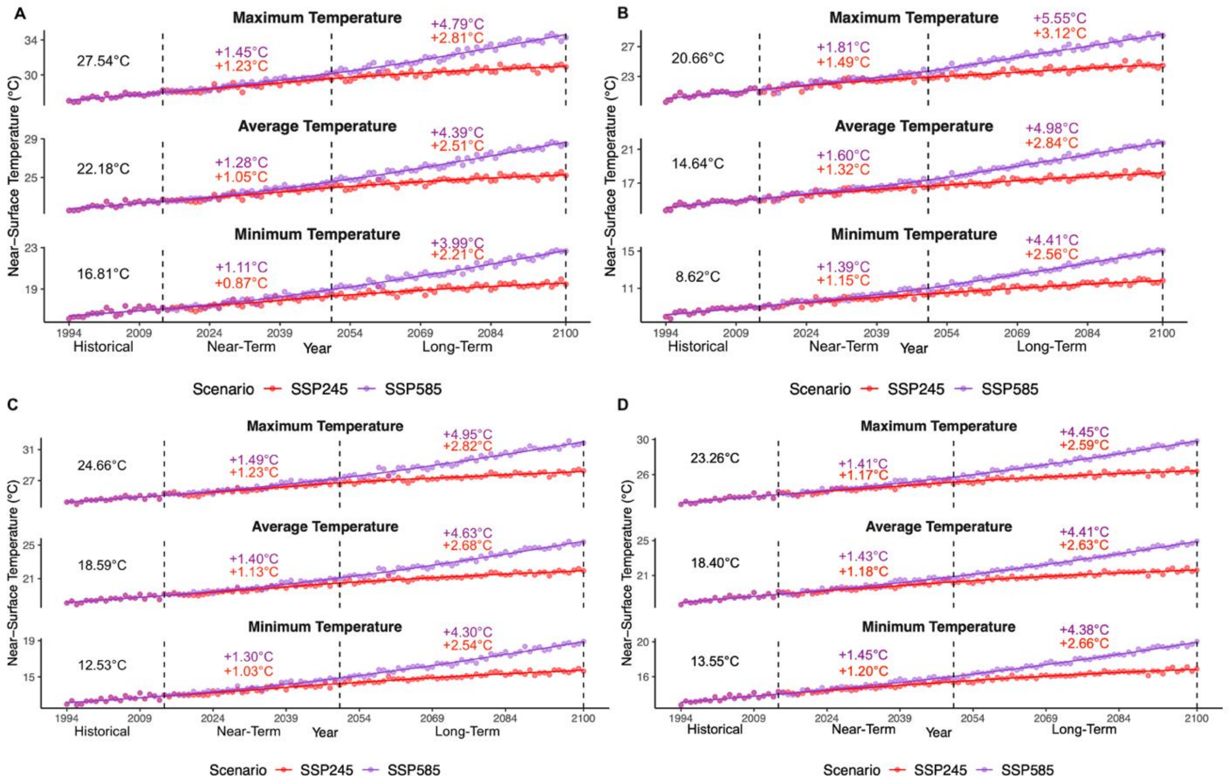

Figure S2: **Flowering Near-Surface Temperature**
Historical, SSP245, and SSP585 multi-model CMIP6 yearly flowering near-surface temperature for A) Ontario, B) British Columbia, C) Quebec, and D) Nova Scotia. Each plot point represents a yearly spatial-temporal average across the region and flowering period. In each plot, a locally estimated scatterplot smoothing (LOESS) curve is fit with a span of 0.75. The annotated values represent the change of near-surface temperature near-term (2015-2050) and long-term (2051-2100) in relation to the historical time period (1994-2014).

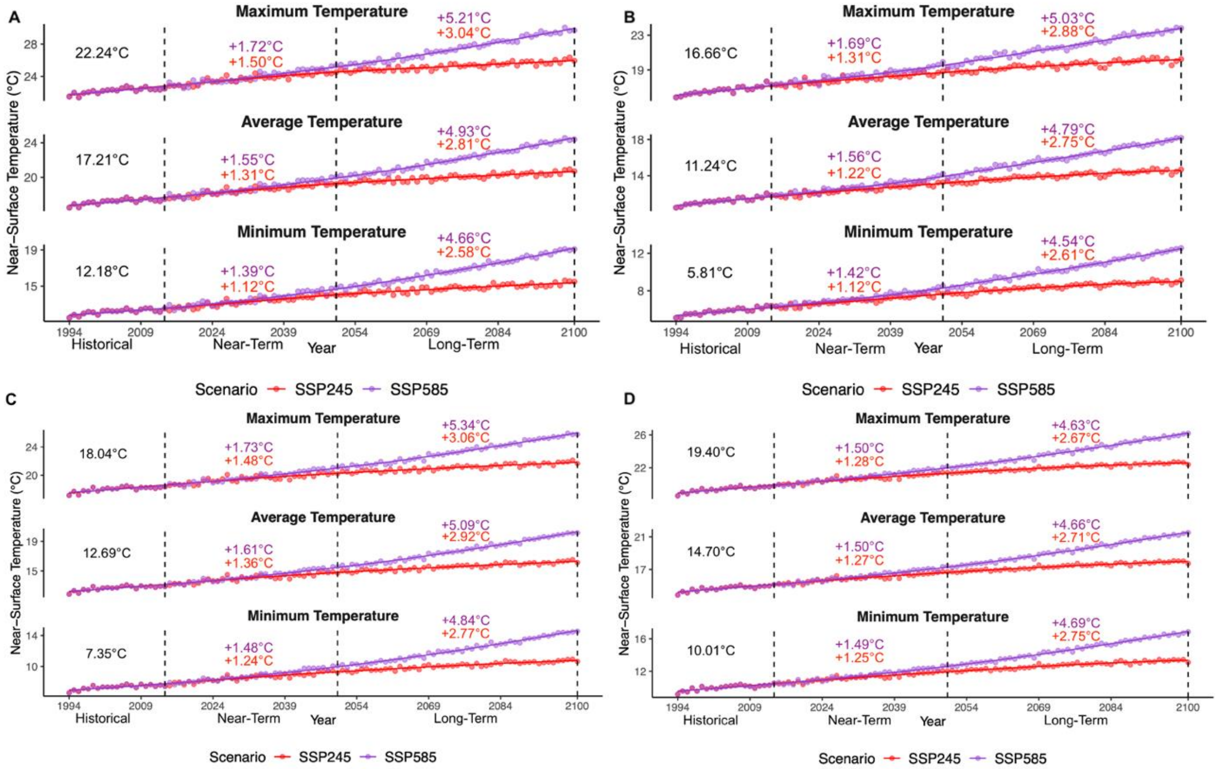

Figure S3: **Veraison/Harvest Near-Surface Temperature**
Historical, SSP245, and SSP585 multi-model CMIP6 yearly veraison/harvest near-surface temperature for A) Ontario, B) British Columbia, C) Quebec, and D) Nova Scotia. Each plot point represents a yearly spatial-temporal average across the region and veraison/harvest period. In each plot, a locally estimated scatterplot smoothing (LOESS) curve is fit with a span of 0.75. The annotated values represent the change of near-surface temperature near-term (2015-2050) and long-term (2051-2100) in relation to the historical time period (1994-2014).

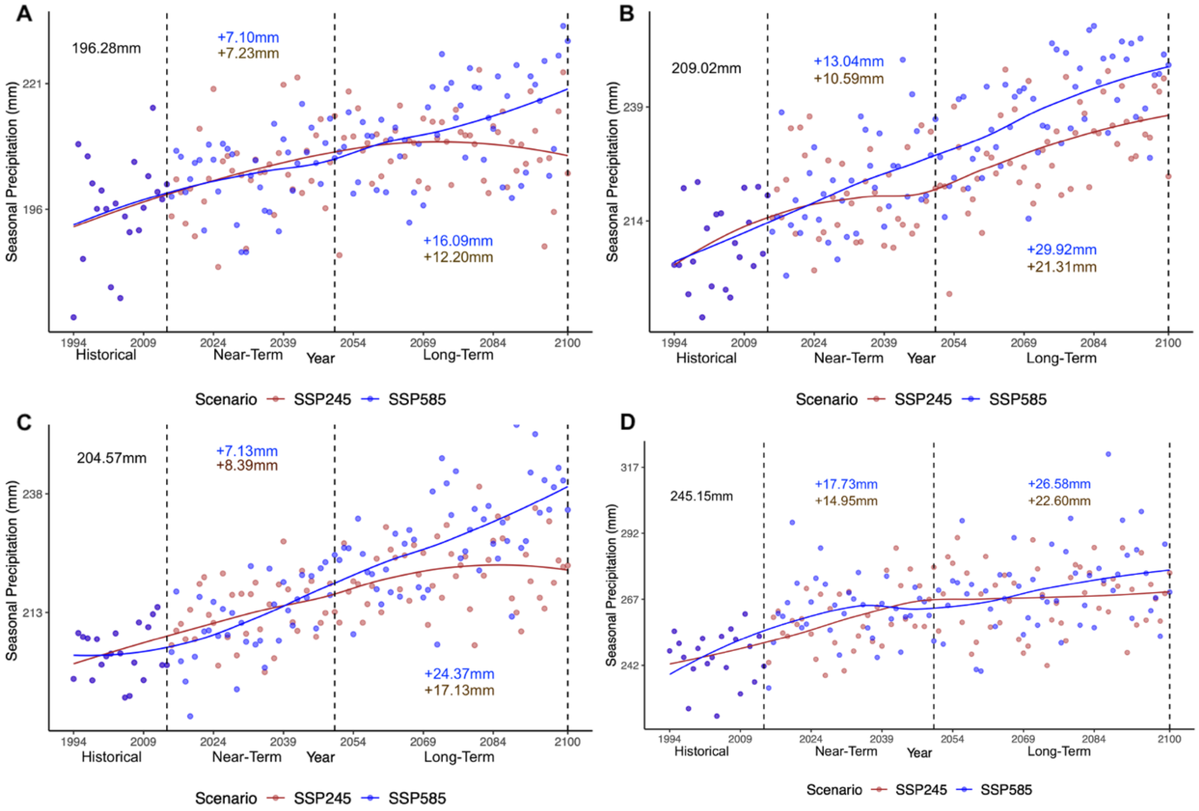

Figure S4: **Bud Burst Seasonal Precipitation**
Historical, SSP245, and SSP585 multi-model CMIP6 yearly bud burst precipitation for A) Ontario, B) British Columbia, C) Quebec, and D) Nova Scotia. Each plot point represents a yearly spatial-temporal average across the region and bud burst period. In each plot, a locally estimated scatterplot smoothing (LOESS) curve is fit with a span of 0.75. The annotated values represent the change of near-surface temperature near-term (2015-2050) and long-term (2051-2100) in relation to the historical time period (1994-2014).

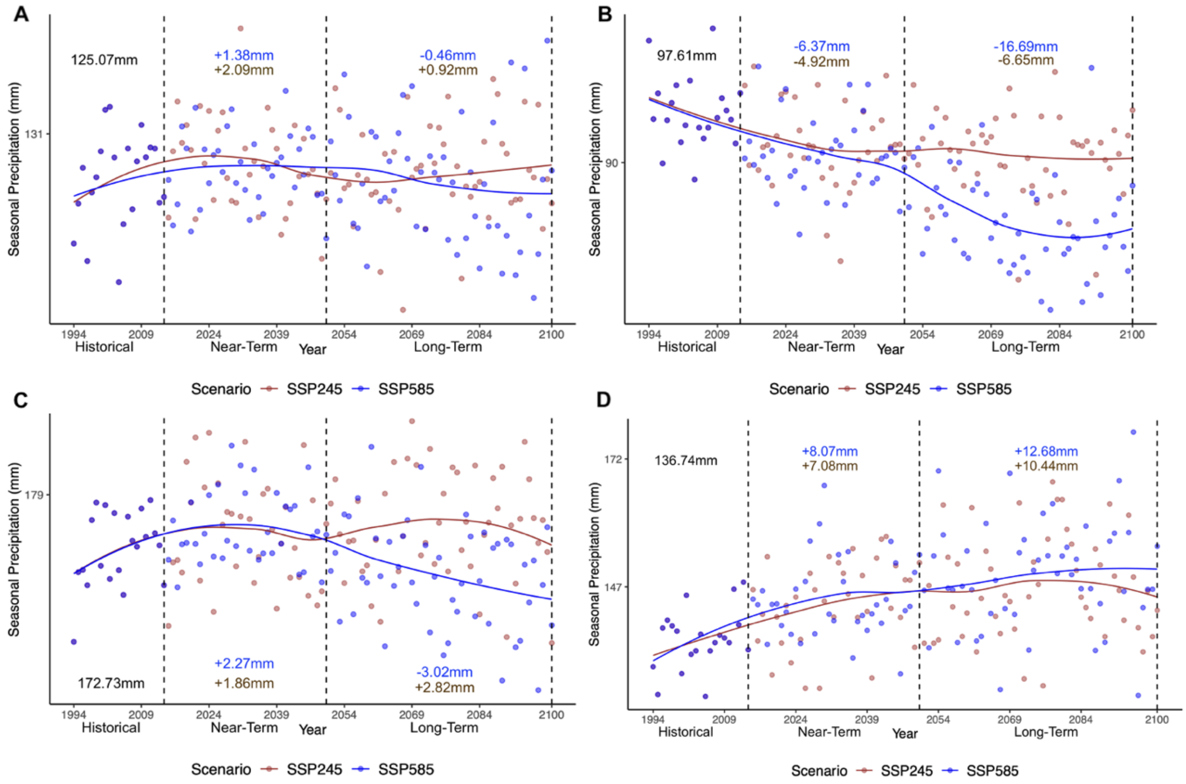

Figure S5: **Flowering Seasonal Precipitation**

Historical, SSP245, and SSP585 multi-model CMIP6 yearly flowering precipitation for A) Ontario, B) British Columbia, C) Quebec, and D) Nova Scotia. Each plot point represents a yearly spatial-temporal average across the region and flowering period. In each plot, a locally estimated scatterplot smoothing (LOESS) curve is fit with a span of 0.75. The annotated values represent the change of near-surface temperature near-term (2015-2050) and long-term (2051-2100) in relation to the historical time period (1994-2014).

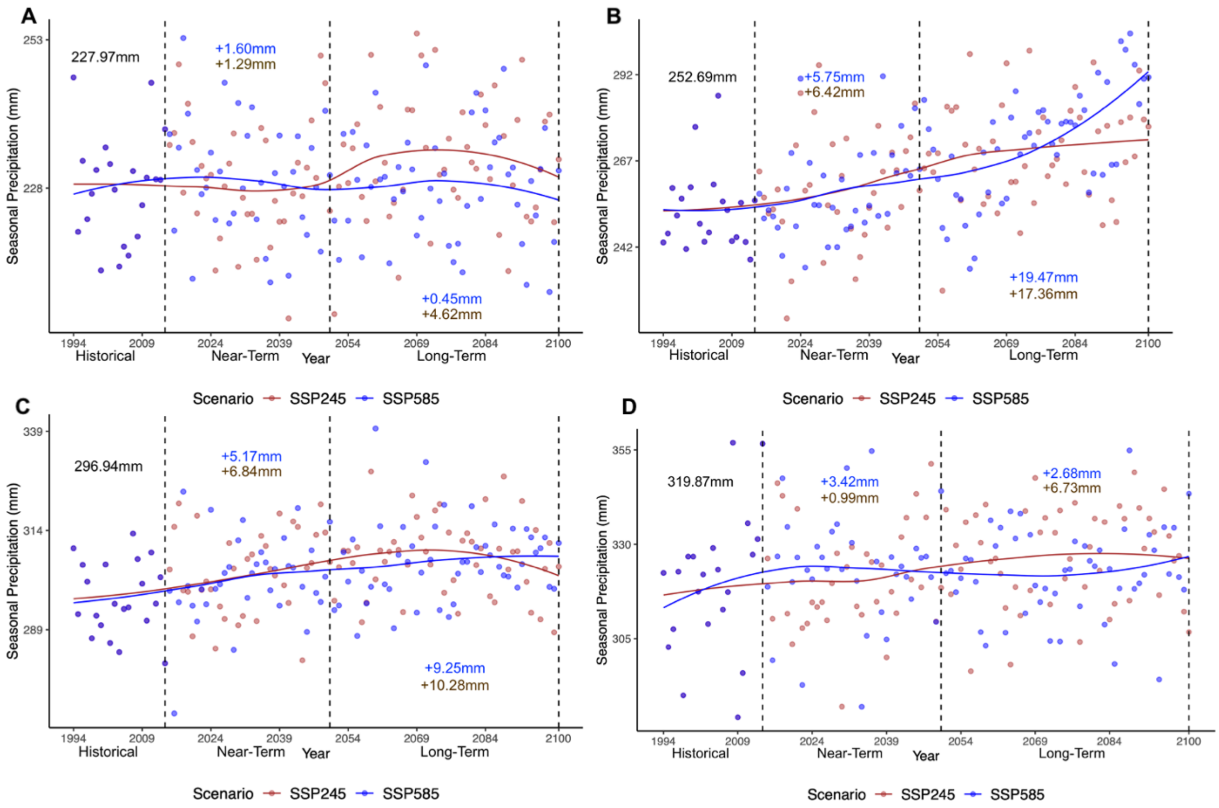

Figure S6: **Veraison/Harvest Seasonal Precipitation**

Historical, SSP245, and SSP585 multi-model CMIP6 yearly veraison/harvest precipitation for A) Ontario, B) British Columbia, C) Quebec, and D) Nova Scotia. Each plot point represents a yearly spatial-temporal average across the region and veraison/harvest period. In each plot, a locally estimated scatterplot smoothing (LOESS) curve is fit with a span of 0.75. The annotated values represent the change of near-surface temperature near-term (2015-2050) and long-term (2051-2100) in relation to the historical time period (1994-2014).
